# Cognitive Differences and the Coding Analysis of the Interaction Behavior Patterns in the Innovation Team

**DOI:** 10.3389/fpsyg.2022.918238

**Published:** 2022-06-10

**Authors:** Yan Zhao, Huangyi Gui, Tianjiao Hu, Ke Xu

**Affiliations:** School of Management, Shanghai University, Shanghai, China

**Keywords:** cognitive differences, perception, cultural psychology, innovation teams, interaction behavior patterns, coding analysis, innovative performance

## Abstract

Despite a wealth of research on the interaction behavior patterns among team members from different angles, few studies focus on the combination of innovation management and innovation team. With the “Input-Process-Output” theoretical framework, this study takes the coding analysis to explore the differences in the interaction behavior patterns of members caused by the cognitive differences in the higher and lower innovative-performing teams. An innovation experiment was conducted in 12 innovation teams based on an experimental paradigm proposed for team innovation tasks. Subsequently, team members’ 1,754 behaviors were coded to analyze the similarities and differences in the interaction behavior patterns between higher and lower innovative-performing teams with lag sequential analysis. The results revealed that both higher and lower innovative-performing teams showed some same interaction behavior patterns. More specifically, the probability of idea facilitation behaviors being followed by team spirit facilitation behaviors was significantly higher than expected, while the probability of idea facilitation behaviors recurring was significantly lower than expected. However, in lower innovative-performing teams, there were some special interaction behavior patterns, such as “the probability of idea facilitation behaviors being followed by neutral interaction or idea inhibition behaviors was significantly lower than expected.” These phenomena may reflect some realistic situations in our life, such as “One echoes the other,” “Sitting on the sidelines” and “A gentleman is ready to die for his bosom friends” in the members’ interaction after cognitive differences happen. This paper provides opinions and suggestions for the research on the interaction behavior observation and coding analysis among members of innovation teams, as well as theoretical contributions to the research on the behavior observation of innovation teams.

## Introduction

Nowadays, it is challenging for someone to discuss with others at the team level because team members’ cognition of others’ ideas and intentions is limited (van Kleef et al., 2017), which results in cognitive differences (Wang and Chin, 2020; Chin et al., 2022). In 1992, Nooteboom first put forward the concept of cognitive difference and believed that it stemmed from the imbalance of cognitive ability between individuals, but individuals could still benefit from different backgrounds or different views held by others through the process of knowledge transfer if the basis of interaction is established (Nooteboom, 1992). In other words, although there are cognitive differences in knowledge and skills among individuals in the team, the team can gather the different ideas of team members (Chin et al., 2021b; Duan et al., 2021) and promote information exchange (Shin et al., 2012; Huang et al., 2022). However, it is unclear whether such differences ultimately promote or hinder team output or performance (Qi et al., 2022), such as the uncertain generation of more extreme success or failure of innovation (Taylor and Greve, 2006). In innovation teams such as R & D teams, excessive cognitive differences among individuals affect team cohesion and decision-making efficiency, resulting in the decline of team innovation performance (Tsui et al., 1992; Smith et al., 1994), but interaction behaviors such as communication among members can help innovation teams promote their innovation performance (Zouaghi et al., 2020; Chin et al., 2021a). The Theory of Interactive Team Cognition points out that the essence of cognitive activities at the team level is the interaction activities among members in the face of dynamic task situations (Cooke et al., 2013). To sum up, the cognitive differences in the cognitive process result in individuals’ different views on different things, and how this difference affects the team’s outcome is closely related to the actual interaction behaviors among team members. The existence of differences in team innovation performance may originate from the interaction behaviors caused by cognitive differences. Therefore, further studying the cognitive process reflected by the interaction behaviors of innovation teams (Vogel and Awh, 2008) and exploring the actual interaction situation of innovation teams with different levels of innovation performance, will be of great significance to the management of innovation teams.

As a new method of research on team behaviors, behavior observation and coding analysis will play an important role in the research of innovation teams. In the 21st century, video recording technology (e.g., mobile phones and computers) and software and hardware of computers develop rapidly, with the improvement of the mathematical methods or methodologies for examining interaction processes and behavior patterns in teams at the time dynamic level (Lehmann-Willenbrock and Allen, 2018). Behavior observation and coding analysis, the quantitative approaches to studying the internal interaction process in the team, have gradually emerged and combined behavioral science with multiple cross-scientific studies, such as psychology (Santangelo et al., 2020), pedagogy (Sun et al., 2021), and management (Meinecke et al., 2017; Lehmann-Willenbrock and Chiu, 2018). However, this approach has a narrow application scope and involves only a few research fields. In the field of innovation management, there is less relevant content that focuses on interaction behavior patterns in innovation teams and links them to cognitive differences. In addition, different from the empirical analysis and qualitative analysis used in traditional research of innovation teams, behavior observation and coding analysis can reliably unify and code the continuous and natural interaction behaviors (Keyton, 2018). Through quantitative analysis methods such as lag sequence analysis, it is possible to explore the systematic behavior patterns in the interaction process of innovation teams to make a more accurate interpretation and inference of collected behavior data (Keyton, 2018).

Over the past decade, some studies have covered the interaction behavior patterns among team members from different angles depending on the lag sequence analysis. Relevant studies can be mainly divided into two directions: First, the study directly explores the characteristics of interaction behavior patterns in the teams. For example, some specific behavior patterns are found in the work team, such as complaining circles (a complaining statement followed by a second complaining statement) and solution circles (a solution-oriented statement followed by a second solution-oriented statement) (Kauffeld and Meyers, 2009; Lehmann-Willenbrock et al., 2015). Second, the study concentrates on the similarities and differences in team members’ interaction behaviors in teams with different levels of performance. For example, during anesthesia inductions, more “non-directed information-non-directed information” behavior patterns are found in higher-performing anesthesia teams, while these behavior patterns have a lower probability of occurrence in lower-performing anesthesia teams (Kolbe et al., 2014). However, most of the previous studies focus on the real workgroup or working environment (such as regular work meetings, medical surgery, etc.). Few studies combine the research of innovation management with innovation teams. Due to the strict confidentiality requirements of innovation teams and the difficulty in acquiring team interaction behavior data, we take the innovation teams composed of students as the research object, and it is efficient to obtain the interactive behavior data of the innovation teams by the experimental method.

Combined with the “Input-Process-Output” theoretical framework, we believe that it is necessary to deeply study how the cognitive differences among innovation team members affect the interaction behavior patterns and how different interaction behavior patterns influence the innovation output. In this study, we first introduce how behavior observation and coding analysis are applied to the research on interaction behavior patterns in innovation teams. Furthermore, an experimental paradigm of team innovation is proposed which was improved by relying on the previous studies to carry out experiments and collect video data. Subsequently, we examine and analyze the interaction behavior patterns of members in innovation teams. This paper aims to uncover the black box of the characteristics of the interaction behaviors in innovation teams at a micro-dynamic level. Overall, this paper intends to discuss the following research questions:

RQ1: When the members of an innovation team complete a task about innovation in the experiment, what specific interaction behavior patterns exist?
RQ2: What are the differences in the interaction behavior patterns occurring among members in innovation teams with different levels of performance?

## Literature Review

### Cognitive Differences and Team Innovation

At present, researchers have conducted some research on cognitive differences and team innovation. Team members often have cognitive differences in ways of thinking, knowledge, skills, values, and beliefs (Dahlin et al., 2005), but various perspectives, ideas, and ways of thinking can be formed to better results in the process of innovation (Shin et al., 2012). Different views and appropriate debate can improve the comprehensiveness of decision-making in the process of team decision-making and finally realize knowledge creation through discussion and interaction (Mitchell et al., 2009). When there is followership in the team, implicit follow cognitive differences harms the followers’ innovation behaviors which suggests that team managers should choose followers who meet the cognitive characteristics of leaders (Liang et al., 2020). More specifically, in the top management teams, the tenure deviation among team members directly affects the cognitive difference, resulting in different views on the firm’s performance and decision-making, but establishing communication can reduce the cognitive difference (Liang and Picken, 2011). Although researchers find that cognitive differences have a certain mechanism for team innovation and consider the interaction scenarios among team members, the relevant research is mainly empirical, which lack the exploration of more micro-interaction processes or specific behaviors among team members.

Based on the existing research and with the “Input-Process-Output” (IPO) theoretical framework, we agree that the personal characteristics of team members are essential input factors that affect the process of team interaction and then influence the final output of the team (such as emergency state and team performance) (Liu et al., 2022). In other words, the input factors and process factors involved in team cooperation affect the output of the team (Carillo and Okoli, 2011). Therefore, we study team cognition and team innovation with the IPO theoretical framework. We regard the cognition, interaction behavior, and innovation performance of team members as the input factor, process factor, and output factor, respectively. Subsequently, we focus on the interaction behavior patterns caused by the cognitive differences of members of innovation teams in the task context and study the impact of different interaction behavior patterns on team innovation performance.

### Interaction Behaviors and Patterns in Innovation Teams

Although few studies aim at the innovative interaction behaviors of team members, team members’ interaction behaviors have already received extensive attention. Some research has used the temporal method to study actual interaction behaviors in the workplace (Zijlstra et al., 2012; Paletz et al., 2016; Meinecke et al., 2017). For example, occurrences of humor and laughter were key behavioral markers when studying humor in group or team interactions (Lehmann-Willenbrock and Allen, 2014). Group mood is inherently an internal affective process with behavioral manifestations (Lehmann-Willenbrock and Allen, 2018), and some verbal interaction sequences are found to have a relationship to group mood (Lehmann-Willenbrock et al., 2011). Currently, the definition of team interaction has not been unified, but researchers have put forward various opinions on different aspects. The majority of researchers support the description that interaction behaviors among team members are task-oriented and give the definition correspondingly. According to Marks et al. (2001), team members’ interaction behaviors are considered to be the process of mutual communication and emotional sustenance under the constraints of the environment and the team’s task goals. The Team Adaptability Theory proposed by Burke et al. (2006) shows that the discussion or communication on the team’s tasks and others’ feedback reflects the team’s response to the external environment, and the implementation of the team’s plans is crucial to the implementation of innovative behaviors. Relying on previous research on team adaptiveness, Lei et al. (2015) emphasized that team interaction behaviors and processes were the reflections of how teams constantly transited behaviors under different workloads (routine or non-routine situations) to quickly match and effectively respond to the dynamic work context.

The interaction behavior patterns in innovation teams can be extended from existing theories, such as team interaction behavior, team interaction process, and the process dynamics method. We support the opinion that the interaction behavior patterns in innovation teams attempt to show the systematic patterns of multiple interaction phenomena that change over time among members of innovation teams (McGrath and Tschan, 2004; Pilny et al., 2016). The core assumption of this model is that innovation teams can exhibit systematic time patterns in the process of members’ interaction. Moreover, the model mainly studies the common changes and accidental events occurring during the interaction over time (e.g., behavior A is often followed by behavior B in the interactions of an innovation team) (Klonek et al., 2019).

### Behavior Observation and Coding Analysis

Depending on the behavior observation research conducted by Frederick W. Taylor and George E. Mayo, researchers have further studied the coding analysis of interaction behaviors, which can be traced back to the sociologist Mildred B. Parten’s research in the 1930s and the social relations scientist Robert F. Bales’s research in the 1950s. The former summarized the development of interaction behaviors among preschool children through behavior observation, and the latter developed a system that analyzed the social interaction in a small group (Keyton, 2018).

Behavior observation and coding analysis is a systematic method for unifying and coding continuous, naturally-occurring interaction behaviors, then making valid interpretations and inferences from the collected data. Coding means assigning a corresponding category of behavior (e.g., code *Coming up with an idea*) to a behavior unit (e.g., a member’s statement in the discussion, “*I think I could build a retractable clothes rack*”) (Endrejat et al., 2019). Waller and Kaplan (2016) summarized the observation process into four components: Data Collection Site, Coding Schemes and Intervals, Coder Selection and Training, and Analysis Focus. However, their research did not emphasize the identification of the research question before starting the study and ignored the importance of data analysis and interpretation of results when highlighting the coding scheme in data preprocessing and coders’ selection and training. Based on the previous research, Klonek et al. (2019) claimed that the process analysis of high-resolution teams involved four steps: Identification of Research Questions, Data Collection and Management, Data Analysis, and Interpretation of Results. Nevertheless, they ignored the complex issues of ethics and data confidentiality that needed to be considered when collecting data (Israel, 2014). They also did not emphasize the impact that data collection and data preprocessing, which have certain independence respectively, may have on the conclusions of the research when performing the process analysis of interaction. At the same time, their research separated Data Analysis with Interpretation of Results instead of unifying them.

**FIGURE 1:**
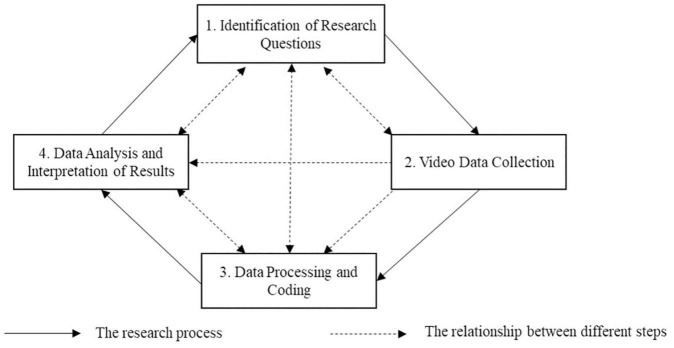
The research processes of behavior observation and coding analysis and the relationship between each step (designed by the authors of this study).

Considering the studies mentioned above, we hold the opinion that the coding analysis of the interaction behavior patterns in innovation teams mainly includes four steps: Identification of Research Questions, Video Data Collection, Data Processing and Coding, and Data Analysis and Interpretation of Results. The four steps are interdependent and mutually restrictive that constitute the framework of the research on the interaction process in innovation teams. Therefore, we design the research processes of behavior observation and coding analysis and the relationship between each step (Figure 1). For example, the research question determines the selection of video data collection scenarios, coding schemes, and data analysis methods. Conversely, the types of video data, coding schemes, and analysis methods limit the breadth and depth of research questions that can be expanded. The quality of video data determines which data can be used for encoding and analysis, and the quality of video data processing and encoding affects the results of data analysis. In contrast, the expected data analysis method influences the choice of the video encoding method.

### Innovative Interaction Behaviors and Innovative Performance

Since experiments on team innovation are usually temporary and fast-paced, the data representing innovative performance mainly originates from the results of the team innovation obtained in the experiment. The majority of the existing research measures innovative performance in two ways. One way is to use the number of innovative solutions proposed by the team in the experiment. For example, Endrejat et al. (2019) tried to use the number of innovative solutions to represent team innovative performance. The other way is to evaluate teams’ innovative achievements or performance by experts’ scoring. At present, scholars generally tend to use the experts’ scores on team innovation results as an indicator to characterize team innovative performance in teams’ interaction behavior experiments (Kolbe et al., 2014; Lehmann-Willenbrock and Allen, 2014; Meinecke et al., 2017).

Existing evidence has proved that team members’ interaction behaviors have an important impact on teams’ innovative performance. Shepperd (1993) pointed out that if team members did not share their information and knowledge or express their own opinions and eventually chose the “free ride”, a negative impact on team performance would occur. Butler (2016) showed that team members’ interaction behaviors such as negotiation and conflict would affect team performance, so it was recommended that team members should strengthen communication to promote collaboration and reduce conflict. Taking the R&D team as an example, some researchers highlighted that the team’s social capital could improve team innovation performance and suggested managers encourage team members to collaborate and communicate with each other to establish a strong social relationship (Zouaghi et al., 2020; Mazzucchelli et al., 2021). However, the majority of the studies above are theoretical analyses or based on questionnaire surveys and have not explored the relationship between the micro-process of interaction behavior and innovative performance in the field of team innovation.

To sum up, by using coding analysis, researchers studied team members’ innovative interaction behaviors and related patterns. They also explored the relationship between the interaction behaviors and teams’ innovative performance, which have accumulated a certain number of results and provided theoretical support for further research. However, there are some deficiencies in the existing research: First, few scholars have conducted a more fine-grained analysis of team members’ innovative interaction behavior patterns from the perspective of microscopic behavioral science through experimental research and other methods. Meanwhile, there is a lack of the experimental paradigm of team innovation in line with reality. Second, previous research on team innovation experiments usually summarizes the phenomena and characteristics captured in the experiments and puts forward relevant hypotheses through theoretical research, but does not consider the more practical and specific behavior characteristics of teams. Therefore, based on the experimental paradigm of team innovation, the combination of experiment, behavior observation, and coding analysis makes it possible to further clarify the patterns and characteristics of team members’ innovative interaction behaviors in team innovation. Simultaneously, establishing and verifying the relationship between the innovative interaction behavior patterns found in the experiment and team innovative performance has certain significance for the management of team innovation.

## Materials and Methods

### Participants

We designed an experiment to carry out a task about team innovation, and video data were collected by video recording. During the experiment, a total of 12 innovation teams (3-4 people in each group) formed by 44 MBA students (24 males and 20 females) participated in this task. The age of participants ranged from 24 to 35 years old (*M* = 28.5, *SD* = 2.77), and the number of females in each group ranged from 0 to 4 people (*M* = 1.67, *SD* = 0.89). All the participants are students enrolled in the MBA program and has obtained a bachelor’s degree. Meanwhile, all of them are employees who work in enterprises or public institutions. In their daily life, they keep in communication with their teammates to complete the team’s tasks and assignments about innovation and entrepreneurship. After taking courses in innovation and entrepreneurship management, they have already had a basic understanding of the theory in the field of innovation and innovation teams.

Before the task, all participants had been informed that the whole process of the team participating in the experiment needed to be recorded. We strictly abide by the privacy and confidentiality regulations and use the video data only for academic research. This study obtained the consent of all participants and had been approved by the Science and Technology Ethics Committee of Shanghai University.

### Experiment Paradigm of Team Innovation Task

When it comes to innovation or creativity experiments, one of the most famous examples is the Alternative Uses Task (AUT) proposed by the American psychologist Joy P. Guilford (1959), which has been widely used in behavioral science research on creativity (Runco and Mraz, 1992; Hao et al., 2017; Xue et al., 2018). At the same time, the Torrance Test of Creative Thinking (TTCT) is also favored by many scholars (Torrance, 1972). In particular, some TTCT experiments, such as the Unusual Uses Task (UUT) or the Picture Completion Task (PCT), have been applied to research individual creativity either separately or collectively (Tang et al., 2018; Nairne et al., 2019; Rominger et al., 2020). However, most of the experimental studies mentioned above concentrate on individual creativity and do not notice the fact that the main source of innovation in current society is the team.

In the field of team innovation, the classic experiment paradigm is brainstorming, proposed by Alex F. Osborn. Brainstorming focuses on the number of participants’ ideas and asks participants not to judge others’ ideas, but they can refer to what other people have expressed to come up with their unique opinions (Osborn, 1953). In recent years, as the research on team innovation has gradually attracted scholars’ attention, Realistic Presented Problem (RPP) tasks that combined with brainstorming have been applied to the research on team innovation, which requires participants to propose as many solutions as possible based on a given realistic problem (Xue et al., 2018; Endrejat et al., 2019).

It is not difficult to find that, no matter which type of task the research uses at the individual level (AUT or TTCT task) or the team level (RPP task), the essence of these experimental tasks is brainstorming or its variants. Moreover, in these tasks creativity is usually measured by the number of ideas proposed by the participants or directly by the ideas’ originality (Endrejat et al., 2019). However, for team innovation, the number of ideas is usually not the most essential element, and the novelty and usefulness of ideas only reflect the level of team creativity (Oldham and Cummings, 1996). When innovation teams conduct innovation activities in reality, they need to focus not only on the generation of ideas but also on the implementation of the ideas (Van de Ven, 1986). Hence, innovation teams should take into account the feasibility of the idea in the stage of idea generation to promote the implementation of the idea, which is the prerequisite for the sustainable development of the innovation team.

Therefore, we proposed an experimental paradigm for team innovation tasks to explore the actual situation of innovation behaviors in team members’ interactions. We required innovation teams to design innovative products under given conditions, and the specific requirements were: Through sufficient discussion and communication, members can use 10 round ABS plastic rods with a diameter of 0.2 cm and a length of 10 cm without any restriction on usage to build an “innovative product” model in 20 minutes. The tools required are readily available, and the links or connection methods are not limited. After the discussion is over or the maximum duration has been reached, they need to draw the “innovative product” model on a given A4 sheet of paper and provide a text description of the product’s innovation.

### Experiment Implementation and Video Data Collection

Before the experiment start, the team members placed the mobile phone in an appropriate position as required and opened the camera of the mobile phone, so that each member could appear in the lens as much as possible. Team members must ensure that the sound can be recorded clearly so that it is convenient to distinguish which member initiated a certain type of interaction behavior. At the same time, all the members were asked to ignore the presence of the mobile phone and carry out the team innovation task naturally. After all the experiments, the time length of the collected video (excluding the time for drawing and filling in text descriptions), which could be used in the analysis, was 100.4 minutes in total, and the duration of experiments in different innovation teams (higher and lower innovative-performing teams) ranged from 4.1 minutes to 20 minutes (*M* = 8.37, *SD* = 5.14).

### Data Processing and Coding

#### Coding Scheme

We adopted the Analyzing Idea Finding Interactions (AIFI) coding system proposed by Endrejat et al. (2019). Endrejat et al. (2019) demonstrated the effectiveness of the AIFI coding system through brainstorming in teams of students and inferred that the coding system could fit into a wider range of creative environments in teams, including traditional brainstorming sessions, team creativity during regular organizational meetings interaction, and team innovation tasks. However, the experiments they designed only reflected the scene of brainstorming when solving a problem and lacked consideration of the nature of innovation.

Despite the distinction between innovation and creativity, the interaction process of innovation teams, including discussions on the feasibility of innovative products and the implementation of ideas, is still a reflection of the interactive discussions on different ideas or viewpoints. AIFI coding system is drawn on the previous literature and related empirical studies in the fields of team creativity, team innovation (Eberle, 1996; Paulus, 2000; van Knippenberg, 2017), and team interaction coding (Waller and Kaplan, 2016; Lehmann-Willenbrock and Allen, 2018). This coding scheme also integrates the group interaction coding system (Bales, 1950; Futoran et al., 1989; Kauffeld et al., 2018) with other coding systems for the idea generation stage (Jackson and Poole, 2003; Sonalkar et al., 2013; Kou and Harvey, 2018). The AIFI coding system divides team innovative interaction behaviors into four categories:

(1) *Idea facilitation behaviors* & *Team spirit facilitation behaviors*: the team is currently moving in the direction of generating and developing new ideas.
(2) *Neutral interaction behaviors*: team members neither support nor hinder the finding of ideas.
(3) *Idea inhibition behaviors* & *Team spirit inhibition behaviors*: team members are moving away from finding new ideas.
(4) *Others*: behaviors cannot be classified into any of the above categories in the coding scheme.

According to Endrejat et al.’s research (Endrejat et al., 2019), there were four categories, which were divided into 15 subcategories. *Idea facilitation behaviors* differentiates four subcategories: *idea expression*, *idea explanation*, *idea development*, and *knowledge*. *Team spirit facilitation behaviors* has two subcategories: *support* and *humor*. *Neutral interaction behaviors* consists of *process organization*, *simultaneous talk*, and *others*. *Idea inhibition behaviors* includes *blocking*, *loss in detail and repetition*, *off-topic conversation*, and *silence*. *Relationship conflict* and *complaining* are two subcategories of *team spirit inhibition behaviors*.

The code *others* has been used in the previous coding schemes for behaviors that cannot be classified into other categories, thereby ensuring the comprehensiveness of the coding scheme that each statement can be coded, such as the research by Kauffeld et al. (2018) and research by Kou and Harvey (2018). The AIFI coding system classifies *others* as neutral interaction behaviors with two subcategories. Although the subcategories of *others* show that the behaviors have no positive or negative impact on team innovation, related behaviors are task-driven. For instance, simultaneous talk means that two or more team members speak at the same time. In the video data collected in our study, when simultaneous talk occurred, different members had the same (e.g., the members’ statements are positive) or different types of speech (e.g., one member makes some positive statements while the other member’s statement is passive). Although these effects are classified as *neutral interaction behaviors* due to the difficulty of measurement, these behaviors are all related to the innovation task. However, some cases in innovation team interactions are entirely unrelated to the innovation task, such as the conversation occurring when someone out of the team wants to borrow a pencil. Therefore, *others* will be regarded as a single category that describes such interaction behaviors to avoid them being involved in the analysis of team interaction behavior patterns. Such a kind of solution is in line with the practice of behavior observation studies where the original coding scheme can be moderately adapted based on the actual situation (Meinecke et al., 2017; Sun et al., 2021). In addition, this coding scheme includes *relationship conflict*, but we did not observe this situation in the actual experiments of our study. Hence, the results of the final data analysis do not contain any content concerning relationship conflict. The specific definitions of each category and the corresponding cases in the video data collected in our study are shown in Table 1.

**TABLE 1:** AIFI coding scheme and cases.

Category	Subcategory	Definition	Case
Idea Facilitation	Idea Expression	Come up with a new idea without further elaboration	“I think we can build a retractable clothes hanger.”
	Idea Explanation	Explain or describe an idea	“It’s like a retractable gate at the entrance to the school, with a crossed diamond in the middle.”
	Idea Development	Develop an idea that has been mentioned before by improving, combining, comparing, or prioritizing it	“It can be made parallel and fixed to the wall for easy storage.”
	Knowledge	Contribute knowledge in a specific area or refer to personal experiences	“It can be fixed with wire, one end of which is connected to the top of the window, and the other end is connected to the front end of a rectangular iron frame protruding from the window.”
Team Spirit Facilitation	Support	Explicitly express the agreement or appreciation with an idea, team member, or the process Ask for further explanation of an idea Ask for advice	“If it’s scalable, it will be more convenient than what we are using in the community.” “What do you mean by that?” “What do you think?”
	Humor	Say something humorously, joke, or laugh	“Haha…”
Neutral Interaction	Process Organization	Remind team members of the remaining time Read the task description Mention the overall task or ask how to continue	“We only have five minutes left.” “We can use 10 round ABS plastic rods with unlimited connection mode.” “Who comes first?”
	Simultaneous Talk	Two or more team members speak at the same time	Team members A and B express their opinions at the same time.
Idea Inhibition	Blocking	Disagree with other team members or express negative feelings	“Your idea is wrong.”
	Loss in detail and repetition	Explain an idea without new information Repeat previous ideas	“I still feel like building a… would be nice.”
	Off-topic Conversation	Statements that do not advance the task of innovation or reflect a lack of interest	“What’s for dinner?”
	Silence	No one speaks for more than six seconds	Innovation interaction is fast-paced and dynamic, and long silences inhibit innovation.
Team Spirit Inhibition	Relationship Conflict	Aggressive speech Sarcastic joke Attempts to undermine the authority or competence of other team members	“You are nothing but a student.” (Endrejat et al., 2019) (Not the case in this article.)
	Complaining	Express disinterest or pessimism Try to find a scapegoat Try to end the discussion as soon as possible	“That does not work.” “It’s all your fault.” “Let’s get this over with.”
Others	Others	Not fit into any of the above categories of interaction	Someone who does not belong to the team asks for a pencil, and a team member of the team replies “Go ahead.”

#### Coding of Interaction Behaviors in Team Innovation

Referring to other team research (Kolbe et al., 2014; Meinecke et al., 2017; Endrejat et al., 2019; Sun et al., 2021), the coding of team members’ innovative interaction behaviors in this study was carried out by two coders who had undergone coding training with experimental videos in advance. Both of them are undergraduate students whose research direction is innovation management.

Different from the INTERACT and The Observer XT software often used in previous studies (for specific software introduction and comparison, see Lehmann-Willenbrock and Allen, 2018), we used the free and open-source behavior observation and research interaction software BORIS 7.10.5 (Friard et al., 2016) to code the interaction behaviors of innovation teams. The BORIS software has been used more than 640 times in the research of animal and human behavior observation in five years (the information came from the Web of Science, up to November 9*^th^*, 2021) and has good reliability. When the coders coded team members’ innovative interaction behaviors, they determined the type of interaction behavior relying on the coding scheme and marked the start time and the end time of each interaction behavior. To determine the consistency, 10% (about 10 minutes) of video data was randomly selected to be coded by the two coders. The inter-rater reliability (IRR) between the two coders was calculated, and Cohen’s κ output by BORIS was 0.718, which indicated a high consistency (Kolbe et al., 2014).

### Team Innovative Performance

To distinguish the innovative performance of innovation teams, we adopted the method in previous studies that experts evaluated the innovative performance (Kolbe et al., 2014; Endrejat et al., 2019). Three experts who specialized in innovation management evaluated the drawings and text descriptions of innovative product models submitted by 12 innovation teams. For the evaluation of innovation, it is generally recognized that innovation needs to reflect novelty and usefulness (Oldham and Cummings, 1996) and should be possible to realize (Van de Ven, 1986; MacCrimmon and Wagner, 1994), so high-quality innovation should take into account all of these factors (Litchfield et al., 2015). We set up three evaluation items: novelty (*“Is it very creative, relatively new?”*), useful (*“Is it very practical or does it have high application value?”*), and feasibility (*“Is it easy to implement in existing or given conditions?”* (Endrejat et al., 2019)). Three experts scored the innovation achievements of 12 innovation teams with a total score of 10 points for each item. The calculation of the intraclass correlation coefficient (*ICC*) was conducted and yielded a value of 0.72 (*p* < 0.001), which indicated good consistency among the three experts. Finally, we took the average of the scores as the final score of the innovative performance of each innovation team.

Based on the previous studies on teams (Stout et al., 1999; Waller, 1999; Waller et al., 2004; Uitdewilligen and Waller, 2018), we used the median split to cluster higher and lower innovative-performing teams. First, based on the data obtained from this study and the independent sample *t*-test method, it was found that higher and lower innovative-performing teams had significantly different scores of innovative performance ((higher innovative-performing teams, *M* = 19.25, *SD* = 3.09; lower innovative-performing teams, *M* = 14.11, *SD* = 0.78), *t*(5.63) = −3.95 (*p* < 0.01)). Therefore, it was effective to use the median split to distinguish them (Kolbe et al., 2014). Second, after sorting teams’ scores from high to low according to the obtained innovation performance scores, the team in the top 50% (the top 6 teams) was defined as a higher innovative-performing team, and the team in the bottom 50% (the bottom 6 teams) was defined as a lower innovative-performing team.

We eventually collected 1754 behavior samples from all members of 12 innovation teams. The descriptive statistics of the higher and lower innovative-performing teams are shown in Table 2.

**TABLE 2:** Descriptive statistics and *t*-test of coded behavior frequency for higher and lower innovative-performing teams.

Category	*N*	Subcategory	*N*	Higher innovative-performing teams			Lower innovative-performing teams			Independent sample t-test	
						
				*M*	*SD*	*N*	*M*	*SD*	*N*	*t*	*p*
Idea Facilitation	595	Idea expression	254	16.50	15.29	99	25.83	18.65	155	0.95	0.37
		Idea explanation	210	13.83	9.77	83	21.17	15.77	127	0.97	0.36
		Idea development	113	9.67	5.75	58	9.17	6.49	55	−0.14	0.89
		Knowledge	18	2.00	2.76	12	1.00	1.26	6	−0.81	0.44
Team spirit Facilitation	668	Support	574	42.17	26.95	253	53.50	38.63	321	0.59	0.57
		Humor	94	3.50	5.17	21	12.17	8.66	73	2.11	0.06
Neutral Interaction	237	Process organization	153	9.83	7.55	59	15.67	9.16	94	1.20	0.26
		Simultaneous talk	84	6.17	5.56	37	7.83	6.62	47	0.47	0.65
Idea Inhibition	190	Blocking	82	5.83	5.46	35	7.83	4.62	47	0.69	0.51
		Loss in detail and repetition	71	4.33	4.55	26	7.50	4.59	45	1.20	0.26
		Off-topic conversation	24	0.50	1.22	3	3.50	3.51	21	1.98	0.08
		Silence	13	0.67	1.21	4	1.50	1.97	9	0.88	0.40
Team Spirit Inhibition	38	Relationship conflict	0	/	/	/	/	/	/	/	/
		Complaining	38	1.17	1.94	7	5.17	6.94	31	1.36	0.20
Others	26	Others	26	2.00	3.16	12	2.33	2.42	14	0.20	0.84

### Lag Sequential Analysis

We used lag sequence analysis (Bakeman and Gottman, 1997) and GSEQ 5.1 (Bakeman and Quera, 2011), a software that analyzes interaction sequence, to find out the members’ interaction behavior patterns in the innovation team. The lag sequence analysis can examine the contingencies and behavior patterns in the events coded based on the sequence of occurrence, and determine the probability of which behavior patterns’ occurrence are significantly higher or lower than expected (Bakeman and Gottman, 1997; Bakeman and Quera, 2011). In this study, BORIS 7.10.5 generated SDS files for analysis in GSEQ 5.1 to obtain two lag 1 interaction sequence transition matrices of higher and lower innovative-performing teams and then determined the transition frequency of each pair of codes in two teams. The adjusted residual (*z-score*) can test whether the transition probability of each pair of codes is significantly different from the unconditional probability of the following codes (Bakeman and Gottman, 1997). The adjusted residual (*z-score*) is calculated as follows:


$$\begin{array}{c} z_{G\,T}=\frac{x_{G\,T}-m_{G\,T}}{\sqrt{m_{G\,T}\,\left(1-p_{G+}\right)\,\left(1-p_{+T}\right)}} \end{array}$$

G stands for a given behavior, and T stands for a target behavior. *z*_*GT*_ refers to the adjusted residual of the frequency of occurrence that given behavior G transits to target behavior T. *x*_*GT*_ and *m*_*GT*_ refer to the observed value and expected value of the frequency of the behavior transition respectively. *p*_*G+*_ and *p*_*+T*_ stand for the probability of the occurrence of given behavior G and target behavior T respectively.

The purpose of calculating the adjusted residual (*z-score*) is to compare the observed value of the behavior transition frequency with its expected value. Subsequently, we determined which transition probabilities significantly deviated from their expected value to verify whether there was a certain transition relationship among behaviors. When the significance level is 5%, if the *z-score* is greater than 1.96, it indicates that the probability of the behavior transition (given behavior G transits to target behavior T) is significantly higher than expected. If the *z-score* is less than -1.96, it indicates that the probability of the behavior transition is significantly lower than expected (Bakeman and Gottman, 1997; Bakeman and Quera, 2011; Lehmann-Willenbrock et al., 2015; Sun et al., 2021).

## Results and Analysis

**FIGURE 2:**
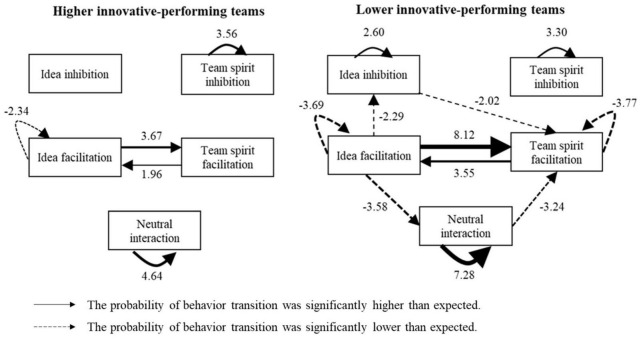
Behavior patterns of higher and lower innovative-performing teams.

First, through the independent *t*-test of a single behavior, the differences of each code’s occurrence in the higher and lower innovative-performing teams were tested (see Table 2). Second, we analyzed the interaction behavior patterns for higher and lower innovative-performing teams (see Table 3). Meanwhile, according to all the behavior sequences which have significantly adjusted residuals (*z-score*) in Table 3, the innovative interaction behavior pattern diagram of higher and lower innovative-performing teams was constructed (see Figure 2, excluding *others*). In Figure 2, arrows indicate behavior patterns or characteristics. Solid lines mean that the probability of transition is significantly higher than expected while dashed lines mean that the probability of transition is significantly lower than expected. Numbers indicate the adjusted residual (*z-score*) of the transition frequency. The thickness of arrows and lines is positively correlated with the adjusted residual (*z-score*).

**TABLE 3:** Comparison of adjusted residuals (*z*-scores) of behavior transitions between higher and lower innovative-performing teams.

		Target Behavior											
		
Given behavior		Idea Facilitation		Team spirit Facilitation		Neutral Interaction		Idea Inhibition		Team Spirit Inhibition		Others	
							
		*Z*	*p*	*Z*	*p*	*Z*	*p*	*z*	*p*	*z*	*p*	*Z*	*p*
Idea Facilitation	HIPT	−**2.34**	0.019	**3.67**	<0.001	−1.94	0.053	−0.10	0.920	0.39	0.697	−0.18	0.855
	LIPT	−**3.69**	<0.001	**8.12**	<0.001	−**3.58**	<0.001	**−2.29**	0.022	−0.86	0.392	−0.92	0.357
Team spirit Facilitation	HIPT	**1.96**	0.050	−0.71	0.478	−1.25	0.211	0.02	0.984	−0.52	0.601	−0.94	0.345
	LIPT	**3.55**	<0.001	−**3.77**	<0.001	−1.28	0.199	1.43	0.152	0.89	0.373	−0.14	0.887
Neutral Interaction	HIPT	−1.38	0.168	−1.90	0.058	**4.64**	<0.001	−0.11	0.915	0.05	0.961	0.31	0.759
	LIPT	−0.63	0.527	−**3.24**	0.001	**7.28**	<0.001	−1.28	0.200	−1.18	0.240	0.08	0.937
Idea Inhibition	HIPT	1.31	0.190	−1.70	0.089	0.75	0.450	0.18	0.855	−0.87	0.384	−0.16	0.874
	LIPT	1.22	0.221	−**2.02**	0.044	−0.34	0.732	**2.60**	0.009	−0.93	0.353	−1.37	0.169
Team Spirit Inhibition	HIPT	1.21	0.227	−1.35	0.178	−1.04	0.299	0.41	0.678	**3.56**	<0.001	−0.35	0.726
	LIPT	−0.07	0.946	−0.65	0.516	−0.06	0.950	−0.36	0.717	**3.30**	0.001	−0.66	0.509
Others	HIPT	1.07	0.287	−1.00	0.317	−1.36	0.172	−0.16	0.874	−0.35	0.726	4.04	<0.001
	LIPT	−1.35	0.178	−1.10	0.270	−0.6	0.550	0.41	0.681	1.00	0.315	9.26	<0.001

*HIPT = Higher innovative-performing teams, LIPT = Lower innovative-performing teams.*

*Bold values mean the probability of the behavior transition is significantly higher (if the z score is greater than 1.96) or lower (if the z score is less than −1.96) than expected.*

### Idea Facilitation Behaviors

After the idea facilitation behavior occurred, the probability of spirit facilitation behaviors’ occurrence was significantly higher than expected in both higher (*z* = 3.67, *p* < 0.001) and lower innovative-performing (*z* = 8.12, *p* < 0.001) teams, but the probability of idea facilitation behaviors’ recurrence was significantly lower than expected (higher innovative-performing teams: *z* = −2.34, *p* = 0.019; lower innovative-performing teams: *z* = −3.69, *p* < 0.001). Compared with higher innovative-performing teams, the probability of neutral interaction behaviors (*z* = −3.58, *p* < 0.001) and idea inhibition behaviors (*z* = −2.29, *p* = 0.022) occurring after idea facilitation behaviors were significantly lower than expected in lower innovative-performing teams.

### Team Spirit Facilitation Behaviors

After the team spirit facilitation behaviors observed in the experiment, the probability of observed idea facilitation behaviors was significantly higher than expected in both higher (*z* = 1.96, *p* = 0.050) and lower innovative-performing (*z* = 3.55, *p* < 0.001) teams. However, lower innovative-performing teams were significantly less likely than higher innovative-performing teams to engage in team spirit facilitation behaviors once more (*z* = −3.77, *p* < 0.001).

### Neutral Interaction Behaviors

After the neutral interaction behaviors occurred in the experiment, the probability of members’ engagement in neutral interaction once more was significantly higher than expected in both higher (*z* = 4.64, *p* < 0.001) and lower innovative-performing teams (*z* = 7.28, *p* < 0.001). Furthermore, members in lower innovative-performing teams were less likely to conduct spirit facilitation behaviors after neutral interaction behaviors (*z* = −3.24, *p* = 0.001).

### Idea Inhibition Behaviors

After the occurrence of idea inhibition behaviors in the experiment, the probability that idea inhibition behaviors recurred in lower innovative-performing teams was significantly higher than expected (*z* = 2.60, *p* = 0.009), while team spirit facilitation behaviors were significantly less likely to occur than expected (*z* = −2.02, *p* = 0.044).

### Team Spirit Inhibition Behaviors

After the occurrence of team spirit inhibition behaviors in the experiment, the probability of team spirit inhibition behaviors’ recurrence was significantly higher than expected in both higher (*z* = 3.56, *p* < 0.001) and lower (*z* = 3.30, *p* = 0.001) innovative-performing teams.

## Discussion

### Idea Facilitation Behaviors

It can be inferred that there are similarities in the behavioral characteristics of higher and lower innovative-performing teams. In both types of teams, after a team member proposed an idea to promote or develop innovation, even if some team members held different views on other people’s ideas, they usually gave inquiries or support but were not eager to provide their insights or improve the idea. Therefore, the interaction behavior pattern “*idea facilitation*–*team spirit facilitation*”, which shows “One echoes the other” rather than “Two members bat back and forth”, is one of the important interaction behavior patterns of team innovation. In addition, there are unique behavioral pattern characteristics in lower innovative-performing teams. When a team member proposed an idea to promote or develop innovation, other members were less likely to exhibit behaviors that were related to the progress of the task or detrimental to the innovation. For the participants, our experiment task was different from their classroom tasks or work related to their courses and academic performance. We did not declare that the experiment task related to their course grades may be an important reason they did not perform positively. There are cognitive differences in the attention to tasks of team members, so some of them expressed low enthusiasm for the mission. One team member did not directly refute content that conflicted with their views and confront other teammates, but chose to “Sitting on the sidelines.” This phenomenon is consistent with the existing research on teams’ innovation climate that an explicit innovation climate is necessary for such a condition. Without an explicit innovation climate in the team, members do not have the motivation and initiative to seek changes and innovation (Chen et al., 2013; Zhu et al., 2016). Therefore, in lower innovative-performing teams, although a member expressed encouragement, admiration, or inquiries on idea facilitation behavior, he held attitudes of “Sitting on the sidelines” and indifference without any constructive comments.

### Team Spirit Facilitation Behaviors

In higher and lower innovative-performing teams, members supported others’ opinions or expressed encouragement or humorous words, which usually elicited interaction behaviors to facilitate ideas. Therefore, the interaction behavior pattern “*team spirit facilitation*–*idea facilitation*” reconfirms the behavioral characteristics “One echoes the other” in team innovation. Turning to the lower innovative-performing teams, one member supported someone’s opinion and inquired for more details, which elicited even fewer team spirit facilitation behaviors. This means that, in contrast to the member who inquires for more details on someone’s viewpoint and shows the cognitive differences, other members in lower innovative-performing teams lack the motivation to continue inquiring, which reflects their attitude of “Sitting on the sidelines” and indifference. In the higher innovative-performing teams, members were not significantly more or less likely to express their encouragement or support when a member had conducted the same behavior (*z* = −0.71, *p* = 0.478). With the behavior pattern in higher innovative-performing teams “*idea facilitation–team spirit facilitation*”, we can find that when members proposed or facilitated ideas have perceived support or approval from other members (even if there are only a few supports), they will gain much intrinsic motivation. So that they will take the initiative and be more willing to contribute to promoting innovation (Chen et al., 2013; Zhu et al., 2016), no matter whether there are more supportive statements from others. Therefore, in higher innovative-performing teams, as long as members receive approval or support from teammates, they will embody an attitude: “A gentleman is ready to die for his bosom friends” and continue promoting creativity to innovation.

### Neutral Interaction Behaviors

We found that the behavior pattern “*neutral interaction–neutral interaction*” occurred in both higher and lower innovative-performing teams. This phenomenon indicates that both dialogs about task process management among members and the behavior of multiple team members speaking will appear continuously, which is one of the common interaction behavior patterns in team innovation activities. However, in lower innovative-performing teams, members typically do not show support, encouragement, and humorous for process organization and simultaneous talk behaviors, which reconfirms the attitude of “Sitting on the sidelines” and indifference among members in lower innovative-performing teams. It proves that the internal motivation of information processing and thinking in knowledge tasks is the key for members of a cognitive diversity team to take advantage of all kinds of resources (Kearney et al., 2009).

### Idea Inhibition Behaviors

Idea inhibition behaviors mainly include behaviors of blocking, loss in detail and repetition, off-topic conversation, and silence. These behaviors usually occur when all team members find one idea is defective or cannot advance in idea facilitation. We found that the behavior pattern “*idea inhibition–idea inhibition*” occurred in the lower innovative-performing team. This phenomenon shows that when the deficiency of a previously proposed idea is found or the team cannot generate a better idea, team members will continue to express their opposition to the previous idea or conduct repetitive and irrelevant dialogue or keep silent. In short, members in lower innovative-performing teams usually do not consciously control the behavior of idea inhibition which result in that this behavior continues to happen. Moreover, team spirit facilitation behaviors in lower innovative-performing teams, such as supporting encouragement, are also less elicited by idea inhibition behaviors, which confirms members’ negative attitudes toward innovation tasks again in lower innovative-performing teams.

### Team Spirit Inhibition Behaviors

Team spirit inhibition behaviors, whether relationship conflict (such as attacking and belittling other members) or complaining (such as sighing), are relatively negative behaviors in team innovation. The results showed that the behavior pattern “team spirit inhibition–team spirit inhibition” occurred in both higher and lower innovative-performing teams. What can be inferred is that the negative emotions experienced by innovation teams while conducting innovation activities are contagious, and there is no difference in this characteristic between teams with different performances.

### Contributions

#### Theoretical Contributions

First, in the past, empirical studies using questionnaires or qualitative research using interviews were often used in studies on teams (Shepperd, 1993; Butler, 2016; Zouaghi et al., 2020; Mazzucchelli et al., 2021), while few scholars paid attention to the team innovation interaction process at the micro-level and experiment paradigm of team innovation. Based on the brief introduction of the research of team behavior observation (theories, methods, and tools) and team interaction process, we improve and propose an experimental paradigm for team innovation tasks to analyze the behavior patterns in the process of team innovation interaction according to the theoretical framework of “Input-Process-Output” in the experimental research. Second, Endrejat et al. (2019) proposed the AIFI coding scheme and believed that it could be applied to a wider range of creative environments in teams. Our study is a testament to the AIFI coding scheme, and we modify this scheme. Subsequently, we use experimental methods to expand the behavior observation coding and lag sequence analysis, which are quantitative analysis methods in the research on teams’ behavior patterns, to the studies of the innovation team. We promote the cross-integration of behavioral science theories and innovation team management theories. Meanwhile, providing some theoretical contributions to the research on team behavior observation, behavior patterns, and innovative teams.

#### Practical Implications

Our study revealed some realistic situations occurring in the interaction process of innovation teams on a time scale. Some more microscopic behavioral phenomena in innovation teams were discovered, which are of theoretical and practical significance to the study of innovation teams and promote the management of innovation talents and teams in enterprises. First, team managers should not interfere excessively with the interaction process between team members. For example, interrupting in talking is often regarded as the embodiment of “impoliteness,” but the generation of creativity is usually uncertain. A good idea may arise from the conflict caused by cognitive differences among team members. Therefore, for the interaction of team innovation, these “impolite behaviors” need to be reasonably utilized instead of stopping directly. Second, team managers should pay attention to the specific interaction behaviors within the innovation team. For example, negative emotions are contagious in both higher and lower innovative-performing teams. In lower innovative-performing teams, members generally show the attitudes of “indifference” and “Sitting on the sidelines”. Therefore, when similar situations happen, the manager should take measures such as guidance and team reorganization to enhance the team innovation atmosphere to create favorable conditions for team innovation. Third, in the process of team innovative interaction, team members should respond to the core interaction behaviors and patterns actively, such as idea facilitation behaviors, and make their contributions to opinions and suggestions on innovation promotion to help the team achieve better results.

### Limitations and Future Research

Although our study has found some interaction behavior patterns and characteristics among the members of innovative teams, there are still some limitations. First, the lag sequence analysis can only indicate the significant characteristics of members’ interaction behaviors in innovation teams in the order of occurrence, but cannot explain whether there is causality among behaviors. At the same time, it cannot reflect whether the differences in the interaction behavior patterns between higher and lower innovative-performing teams have a causal relationship with innovative performance. In the future, other methods such as regression analysis (Lehmann-Willenbrock et al., 2015; Meinecke et al., 2017) can be used to analyze the relationship between interaction behaviors and innovative performance. Second, all the video data collected in our study originated from MBA students who composed the 12 innovation teams, so the results only reflected the interaction behavior of innovation teams in our experiment. However, based on the suggestion by Klonek et al. (2019), research on more realistic teams may accurately reflect the specifics of team interaction behavior.

## Conclusion

In this study, we observed and coded the behavior patterns of 12 innovative teams for the innovation interaction. Applying the lag sequential analysis method, we captured different innovation interaction behavior patterns in higher and lower innovative-performing teams. Several interesting phenomena were found (see Figure 2).

First, the two clusters of teams have commonalities in innovative interaction behavior patterns or characteristics. (1) When members of innovation teams put forward ideas and contribute to developing others’ ideas or innovations, other members usually actively agree rather than eagerly expressing their different or unique ideas or opinions. After such an agreement occurs in innovation teams, the interaction behaviors that promote creativity or innovation will appear again. Therefore, “One echoes the other” is an important interaction behavior pattern in the innovation team and the main behavior characteristic of team members to deal with cognitive differences and promote the innovation process. (2) Conversation about the progress of a task or simultaneous speeches followed by the same type of behaviors is the same as common sense. (3) When team members express disinterest or pessimism and try to find a scapegoat or end discussions as quickly as possible, similar behaviors will recur. The evidence for this phenomenon is found not only in higher innovative-performing teams but also in lower innovative-performing teams, which is an unexpected finding. This phenomenon may be related to the fact that the tasks in our experiments have no effect on the interests of the participants. Team members have different perceptions of the importance of the task, and some of them will show negative attitudes and implement corresponding behaviors.

Second, the lower innovative-performing teams have some special innovative interaction behavior patterns or characteristics. (1) When team members propose ideas to promote and develop innovation, other members will exhibit fewer task progress or humorous behaviors. Meanwhile, behaviors that inhibit the development of innovations appear less frequently. (2) After team members engage in team spirit facilitation, such as support, encouragement, and inquiry, they will be significantly less likely to continue conducting similar behaviors. (3) Team members typically do not show support, encouragement, or humorous for process organization and simultaneous talk behaviors. (4) Idea inhibition behaviors are more likely to be followed by idea inhibition behaviors. But few team spirit facilitation behaviors (e.g., support, encouragement, and humorous dialog) occur after idea inhibition behaviors. Hence, lower innovative-performing teams often show no more behaviors that are conducive to idea facilitation after echoing idea facilitation behaviors. This phenomenon shows that the members of lower innovative-performing teams may not contradict each other on the surface. However, they are indifferent or behave in the manner of “Sitting on the sidelines”. Besides, they may not be motivated to promote innovation due to a lack of inspiration or knowledge, which is also not beneficial to team innovation.

## Data Availability Statement

The raw data supporting the conclusions of this article will be made available by the authors, without undue reservation.

## Ethics Statement

The studies involving human participants were reviewed and approved by Science and Technology Ethics Committee of Shanghai University. The patients/participants provided their written informed consent to participate in this study.

## Author Contributions

YZ contributed to the conceptualization, experiment design, results interpretation, and reviewing and editing the manuscript. HG contributed to the conceptualization, results interpretation, and reviewing and editing the manuscript. TH contributed to the methodology, experiment design and implementation, statistical analysis, and results interpretation. KX contributed to the methodology, results interpretation, and reviewing manuscript. All authors contributed to the manuscript and approved the submitted version.

## Conflict of Interest

The authors declare that the research was conducted in the absence of any commercial or financial relationships that could be construed as a potential conflict of interest.

## Publisher’s Note

All claims expressed in this article are solely those of the authors and do not necessarily represent those of their affiliated organizations, or those of the publisher, the editors and the reviewers. Any product that may be evaluated in this article, or claim that may be made by its manufacturer, is not guaranteed or endorsed by the publisher.
